# PPL2 Translesion Polymerase Is Essential for the Completion of Chromosomal DNA Replication in the African Trypanosome

**DOI:** 10.1016/j.molcel.2013.10.034

**Published:** 2013-11-21

**Authors:** Sean G. Rudd, Lucy Glover, Stanislaw K. Jozwiakowski, David Horn, Aidan J. Doherty

**Affiliations:** 1 Genome Damage and Stability Centre, University of Sussex, Brighton BN1 9RQ, UK; 2 London School of Hygiene & Tropical Medicine, Keppel Street, London WC1E 7HT, UK

## Abstract

Faithful copying of the genome is essential for life. In eukaryotes, a single archaeo-eukaryotic primase (AEP), DNA primase, is required for the initiation and progression of DNA replication. Here we have identified additional eukaryotic AEP-like proteins with DNA-dependent primase and/or polymerase activity. Uniquely, the genomes of trypanosomatids, a group of kinetoplastid protozoa of significant medical importance, encode two PrimPol-like (PPL) proteins. In the African trypanosome, PPL2 is a nuclear enzyme present in G_2_ phase cells. Following PPL2 knockdown, a cell-cycle arrest occurs after the bulk of DNA synthesis, the DNA damage response is activated, and cells fail to recover. Consistent with this phenotype, PPL2 replicates damaged DNA templates in vitro, including templates containing the UV-induced pyrimidine-pyrimidone (6-4) photoproduct. Furthermore, PPL2 accumulates at sites of nuclear DNA damage. Taken together, our results indicate an essential role for PPL2 in postreplication tolerance of endogenous DNA damage, thus allowing completion of genome duplication.

## Highlights

• Trypanosomatids contain two archaeo-eukaryotic primase-polymerase-like proteins
• PPL2 is essential in the pathogenic bloodstream form African trypanosome
• PPL2 suppresses DNA damage and allows completion of chromosomal replication
• PPL2 mediates translesion DNA synthesis

## Graphical Abstract

**Figure:**
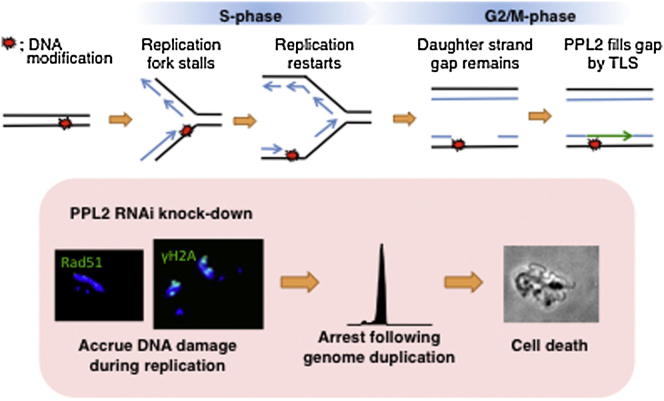

## Introduction

Accurate and complete DNA replication prior to cell division is essential for genome stability. Responsible for this formidable task is a molecular machine containing highly accurate and processive DNA-dependent DNA polymerases. In eukaryotes this role falls to the B-family DNA polymerases, alpha (α), delta (δ), and epsilon (ε). Pol α and its associated DNA primase initiate strand synthesis at replication origins and Okazaki fragments, and Pol δ and Pol ε extend this nascent DNA and replicate the bulk of the genome with high fidelity (Garg and Burgers, 2005). Associated with high fidelity is an inability to synthesize DNA from damaged templates. Indeed, stalling occurs when the replication machinery encounters DNA distortions or modified bases that can result from either environmental insults or endogenous processes. If not resolved, stalled replication forks pose a major threat to genome stability (Aguilera and Gómez-González, 2008). One solution, existing in all domains of life, is translesion synthesis (TLS). Using specialized DNA polymerases capable of directly synthesizing DNA opposite replication fork-blocking lesions, TLS allows the complete replication of the genome in spite of the lack of a pristine DNA template. In eukaryotes the Y-family polymerases eta (η), iota (ι), kappa (κ), and REV1 and the B-family polymerase zeta (ζ) are largely responsible for this process, each with varying capabilities to bypass different DNA lesions (Waters et al., 2009; Sale et al., 2012).

*Trypanosoma brucei*, the African trypanosome, along with the closely related *Trypanosoma cruzi* and *Leishmania’s*, are protozoan parasites of significant medical importance. *T. brucei* is the causative agent of human African trypanosomiasis, a typically fatal condition that is endemic in sub-Saharan Africa (Brun et al., 2010). In addition to their medical importance, trypanosomatids present a unique evolutionary perspective, as they are among the earliest diverging organisms from the eukaryotic tree. As a result, these parasites display a number of features considered unusual among eukaryotes. The major components of the replication machinery in higher eukaryotes have been identified in trypansomatid genomes (El-Sayed et al., 2005), but our understanding of trypansomatid nuclear genome replication (Li, 2012; Tiengwe et al., 2012b) remains far from complete. Conventional DNA repair pathways operate in trypanosomatids (Passos-Silva et al., 2010). However, consistent with early divergence, the replication initiation machinery resembles that of archaea (Godoy et al., 2009; Tiengwe et al., 2012a) and the nonhomologous end-joining break repair pathway does not appear to operate (Burton et al., 2007; Glover et al., 2008).

DNA primases are DNA-dependent RNA polymerases specialized in synthesizing short RNA molecules called primers, which are absolutely required by DNA polymerases to initiate DNA strand synthesis (Frick and Richardson, 2001; Kuchta and Stengel, 2010). In eukaryotes, DNA primase 1 (Prim1) is a member of the archaeo-eukaryotic primase (AEP) superfamily (Iyer et al., 2005) and the catalytic component of the DNA primase associated with Pol α (Muzi-Falconi et al., 2003). In archaea and prokaryotes, AEPs have been shown to be versatile nucleotidyl transferases (Lao-Sirieix et al., 2005) and in some bacteria are required for repair of DNA breaks (Della et al., 2004; Pitcher et al., 2007). *T. brucei* have at least two additional AEPs, both shown to function in mitochondrial (kinetoplast) DNA replication (Hines and Ray, 2010, 2011). The human gene *CCDC111*, encoding for the coiled-coil domain containing 111 (CCDC111) protein, was previously reported to be a putative member of the AEP superfamily (Iyer et al., 2005). In keeping with primase nomenclature and to reflect the intrinsic activities of this AEP, CCDC111 was renamed PrimPol (Primase-Polymerase). Here, we use a divergent eukaryotic model organism to understand the function of PrimPol-like proteins (PPLs). Using iterative PSI-BLAST searches, we identified two PPLs in trypanosomatids and called the *T. brucei* homologs TbPPL1 and TbPPL2; TbPPL2 was essential in bloodstream form *T. brucei*. A combination of molecular, cell biology, and biochemical analyses indicated a role for TbPPL2 in the postreplication tolerance of naturally occurring DNA damage using its TLS activity. Taken together, our results reveal an AEP polymerase required for the completion of nuclear genome duplication.

## Results

### *T. brucei* Genomes Encode Two PPLs

**Figure 1:**
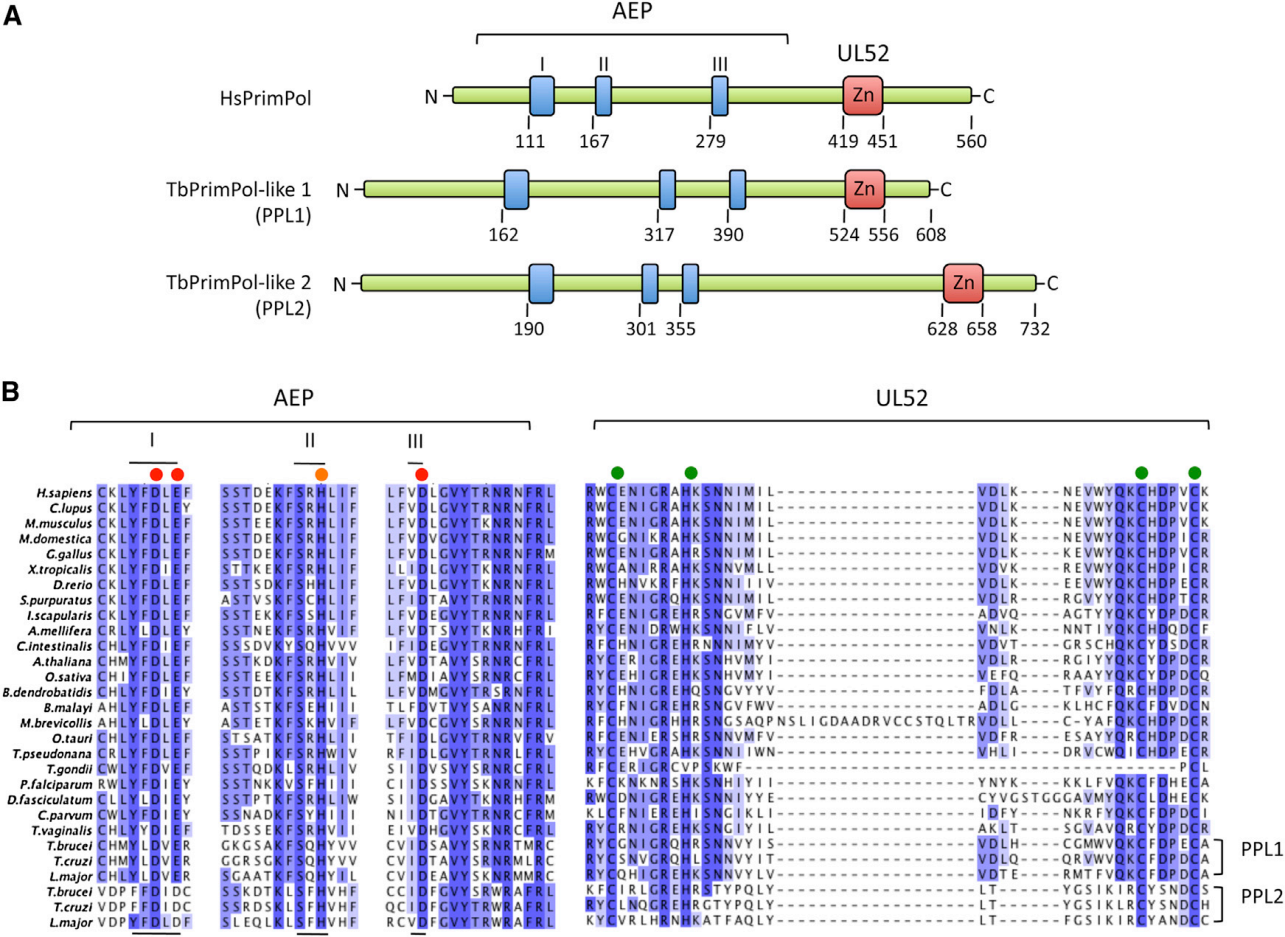
Two PrimPol-like Proteins in Trypanosomatids (A) Schematic showing the domain organization of human and *T. brucei* PrimPols. The highly conserved motifs I, II, and III, comprising the catalytic AEP domain, and the zinc finger with homology to the herpesviral UL52 primase are shown. (B) Multiple sequence alignments of PrimPol-like proteins from a broad range of multicellular and unicellular eukaryotes. Essential residues in the AEP domain are indicated, residues in motifs I and III (red circle) required for magnesium ion binding, motif II (orange circle) for nucleotide binding. The zinc finger motif is also indicated (green circles). See also Figures S1 and S2.

An iterative PSI-BLAST search (Altschul et al., 1997) of the available eukaryotic genomes, using human PrimPol as query, identified PPLs from vertebrates to protists. PPLs were not identified in *Drosophila*, *Caenorhabditis elegans*, and all but one fungus, the parasitic *Batrachochytrium dendrobatidis*. Interestingly, trypanosomatids, the earliest diverging eukaryotes that are readily amenable to genetic manipulation, contain two genes encoding PPLs. Thus, trypanosomatids appear to represent an excellent model system in which to characterize PPLs. We termed the *T. brucei* homologs TbPPL1 (Tb927.5.4070) and TbPPL2 (Tb927.10.2520). TbPPL1 and TbPPL2 share ∼16% and ∼11% identity with human PrimPol, respectively, and share ∼10% identity with each other. Both TbPPL1 and TbPPL2 contain the characteristic domains of the PrimPol family: an N-terminal AEP domain containing the three signature catalytic motifs (I, II, and III) found in all AEP family members and, at the C terminus, a CHC_2_ zinc finger with homology to the herpes viral UL52 primase (Figures 1A and 1B, full alignments in Figures S1 and S2). Notably, TbPPL2 does not contain the variation in catalytic motif I of the AEP domain found in PrimPol family members (DxE), but rather resembles the majority of AEP family members, containing DxD (Figure 1B).

### PPL2 Is an Essential Nuclear Protein in Bloodstream Form *T. brucei*

**Figure 2:**
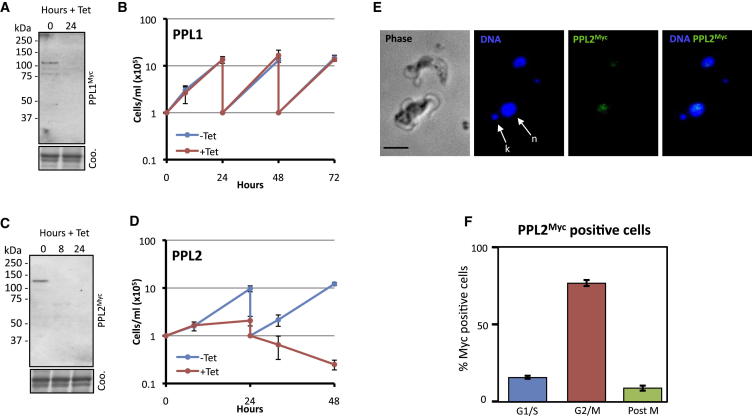
TbPPL2 Is Essential and Cell-Cycle Regulated (A–D) Western blot analysis is shown (A and C) with anti-cMyc and cell lysates prepared from representative TbPPL1^Myc^ and TbPPL2^Myc^ RNAi cell lines, respectively, following RNAi induction with tetracycline (1 μg/ml) for the times indicated. Equivalent Coomassie-stained gels serve as loading controls. Growth curves are shown (B and D) of TbPPL1^Myc^ and TbPPL2^Myc^ RNAi cell lines, respectively, grown in the presence or absence of tetracycline (1 μg/ml). Cultures were diluted to 10^5^ cells/ml every 24 hr as appropriate. Representative growth curves are shown for four experiments performed in duplicate, each with independent cell lines (three without native-tagging). Error bars represent one standard deviation. (E) Immunofluorescent detection of TbPPL2^Myc^ with anti-cMyc. DNA was counterstained with DAPI. Nucleus (n), kinetoplasts (k). Scale bar is 5 μm. (F) Cells with TbPPL2^Myc^ detectable by immunofluorescence were scored for cell-cycle position. G_1_/S, 1 nucleus and 1 kinetoplast (1n1k); G_2_, 1 nucleus and 2 kinetoplasts (1n2k); post-M, 2 nuclei and 2 kinetoplasts (2n2k). n = 200; error bars, standard deviation.

To determine the role of PPL1 and PPL2, we first used inducible RNA interference (RNAi) in bloodstream form *T. brucei*. For the purpose of monitoring protein knockdown following RNAi induction, and to determine subcellular localization, Myc tags were integrated at PPL1 and PPL2 chromosomal loci in the cognate RNAi strains, allowing expression of the C-terminally tagged proteins (PPL1^Myc^ and PPL2^Myc^) under their native transcriptional control. PPL1^Myc^ has a predicted molecular mass of 85.6 kDa (68 kDa without tag) and migrated as a species with an apparent mass of approximately 100 kDa when visualized by western blot analysis (Figure 2A). Twenty-four hours after addition of tetracycline to the culture media of PPL1 RNAi strains, a substantial reduction of PPL1^Myc^ was observed (Figure 2A). Knockdown was not associated with a growth defect even after 72 hr (Figure 2B). PPL2^Myc^ has a predicted molecular mass of 99.4 kDa (82 kDa without tag) and migrated as a species with an apparent mass of approximately 120 kDa (Figure 2C). RNAi knockdown was also efficient in this case (Figure 2C), but in contrast to PPL1, knockdown of PPL2 resulted in a severe growth defect. This defect was clearly visible after 24 hr and was cytocidal thereafter (Figure 2D). We conclude that PPL2 is essential in bloodstream form *T. brucei*, while PPL1 appears to be dispensable.

We next looked at PPL2 subcellular localization using immunofluoresence microscopy. PPL2^Myc^ was detected in the nuclei of cells (Figure 2E). However, it was only detected in a subpopulation of nuclei, prompting the question of whether PPL2 expression is cell-cycle regulated. Cell-cycle stage can be determined in an unperturbed African trypanosome population since the mitochondrial genome (kinetoplast) is visible by DAPI staining and divides in a cell-cycle-dependent manner, preceding nuclear mitosis. Thus, a cell with one nucleus and one kinetoplast (1n1k) represents G_1_/S, one nucleus and two kinetoplasts (1n2k) represents G_2_, and two nuclei and two kinetoplasts (2n2k) represents post-mitosis but pre-cytokenesis (Woodward and Gull, 1990; Siegel et al., 2008). Immunofluorescent detection of PPL2^Myc^ was predominantly (>70%) in G_2_ cells (Figure 2F), which constitute only ∼15% of an asynchronous population. Although recombinant tags sometimes interfere with protein function, this suggested that specific expression control was retained for PPL2^Myc^. This is a striking result for an enzyme involved in DNA synthesis given that DNA replication is advanced, or almost complete, in these cells. Indeed, this suggests a late- or post-DNA replication role. Attempts to determine the subcellular localization of PPL1^Myc^ using immunofluorescence microscopy were unsuccessful (data not shown).

### TbPPL2 Functions Late in DNA Replication

**Figure 3:**
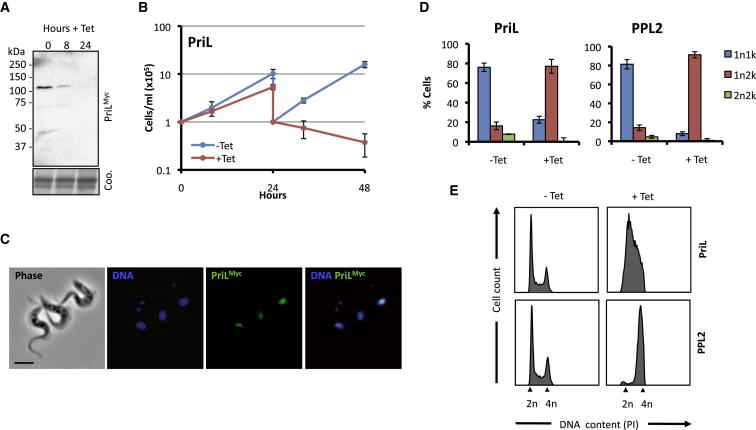
TbPPL2’s Role Is Distinct from the Replicative DNA Primase (A) Western blot analysis with anti-cMyc and cell lysates prepared from a representative TbPriL^Myc^ RNAi cell line following RNAi induction with tetracycline (1 μg/ml) for the times indicated. The Coomassie-stained gel serves as a loading control. (B) Growth curve of TbPriL^Myc^ RNAi cell lines in the absence or presence of tetracycline. Cultures were diluted to 10^5^ cells/ml every 24 hr as appropriate. The growth curve shown is from three experiments performed in duplicate, each with independent cell lines. Error bars represent one standard deviation. (C) Immunofluorescent detection of TbPriL^Myc^ with anti-cMyc. DNA was counterstained with DAPI. Scale bar is 5 μm. (D) Cell cycle distribution of TbPPL2 and TbPriL RNAi cell lines grown in the presence or absence of tetracycline (1 μg/ml for 24 hr). DNA was counterstained with DAPI, and nuclear (n) and kinetoplast (k) counts were scored. Experiments were performed with three independent cell lines for TbPPL2, and two independent cell lines for TbPriL. Error bars indicate one standard deviation. Other details as in legend to Figure 2F. (E) DNA content of TbPPL2 and TbPriL RNAi cell lines grown in the presence or absence of tetracycline (1 μg/ml for 24 hr). Cells were fixed and stained with PI, and DNA content was analyzed by flow cytometry. Representative data are shown for experiments performed with three independent cell lines for TbPPL2 and two independent cell lines for TbPriL.

Since we identified an AEP with an essential function residing in the nucleus, we decided to compare PPL2 and the canonical replicative DNA primase, required for synthesis of RNA primers needed to initiate DNA replication. Throughout eukaryotes and archaea, this is a role ascribed to the heterodimeric DNA primase, composed of a small catalytic subunit (Prim1 or PriS) and large accessory subunit (Prim2 or PriL), which in eukaryotes exist in a complex with Pol α (Pol α-Prim) (Muzi-Falconi et al., 2003). As expected, the *T. brucei* genome encodes a putative DNA primase (El-Sayed et al., 2005). RNAi strains with a native allele tagged, as described above, were assembled for the gene encoding the large subunit (Tb927.10.3110), which we refer to as TbPriL, in keeping with archaeal nomenclature, since a kinteoplastid-specific primase has been named Pri2 (Hines and Ray, 2011). PriL^Myc^ has a predicted molecular mass of 82.8 kDa (65 kDa without tag) and migrated predominantly as a species with an apparent mass of ∼100 kDa (Figure 3A) and, as expected, localized to nuclei (Figure 3C). RNAi knockdown of PriL (Figure 3A) resulted in a severe growth defect (Figure 3B) that was ultimately cytocidal.

We next investigated whether the growth defect that followed PPL2 or PriL knockdown was associated with aberrant cell-cycle progression. As previously described, a useful cytological tool in *T. brucei* is the division of the mitochondrial genome. In uninduced cultures the cell-cycle distribution was as expected in an asynchronous population; ∼80% of cells were 1n1k (G_1_/S), ∼15% were 1n2k (G_2_), and approximately 5% were 2n2k (post-M). However, following knockdown of either PPL2 or PriL, the number of 1n1k cells was reduced to 10%–20%, and the number of 1n2k cells increased to 80%–90% (Figure 3D). This striking cell-cycle arrest following only 24 hr RNAi indicates essential roles for both PPL2 and PriL in *T. brucei* cell-cycle progression.

Accumulation of 1n2k cells is consistent with a defect in DNA replication. To identify the nature of this defect, we next analyzed the DNA content of the cells before and after knockdown. In uninduced cultures of PPL2 and PriL RNAi strains, the cell-cycle distribution was consistent with that of unperturbed cells (Figure 3E). Twenty-four hours of PriL knockdown arrested cells in S phase, preventing them from fully duplicating their genome (Figure 3E). This result is consistent with the established function of the replicative DNA primase from other eukaryotes and suggests that the role of this protein is conserved in trypanosomatids. In contrast, 24 hr of PPL2 knockdown resulted in almost all cells stalling with 4n DNA content (Figure 3E). These data demonstrate that PPL2 is performing a distinct role from known eukaryotic AEPs. PPL2 is not required for initiation and progression of S phase, as knockdown cells can efficiently duplicate the majority, if not all, of their DNA. However, these cells arrest prior to mitosis in late S/G_2_. This phenotype corresponds with our earlier observation that PPL2 accumulates in G_2_ cells (Figure 2F) and indicates that PPL2’s essential function occurs after the bulk of DNA synthesis.

### TbPPL2 Knockdown Triggers Assembly of DNA Damage Signaling and Repair Foci

**Figure 4:**
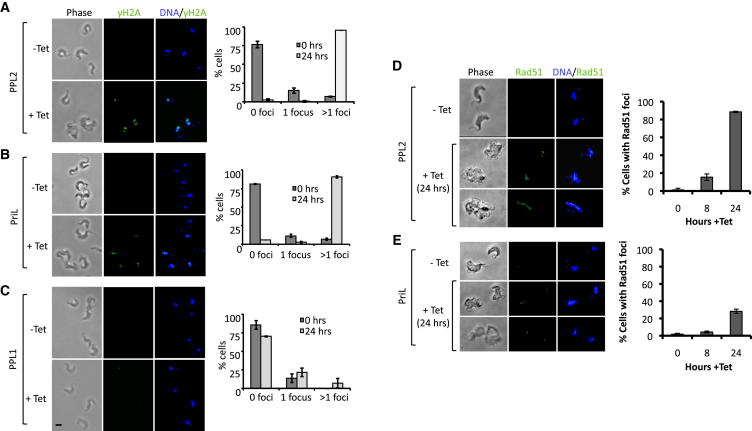
RNA Interference of TbPPL2 Triggers the Focal Accumulation of DNA Signaling and Repair Proteins (A–C) Immunofluorescent detection of γH2A in TbPPL2, TbPriL, and TbPPL1 RNAi cell lines, grown in the presence or absence of tetracycline (1 μg/ml for the time indicated). Representative images including phase-contrast and DAPI-stained DNA are shown. The proportion of cells with nuclear foci were counted (n = 200) and error bars represent one standard deviation. Scale bar is 5 μm. (D and E) Immunofluorescent detection of Rad51 in TbPPL2 and TbPriL RNAi cell lines. Other details as above.

We next examined whether the cell-cycle arrest in *T. brucei* PPL2 and PriL knockdown cells coincides with the accumulation of damaged DNA, as this could explain cell-cycle arrest. A well-known phenomenon is the assembly of DNA repair and signaling proteins into foci, visible by immunofluorescence microscopy at sites of DNA damage. One such signaling protein is the phosphorylated histone (variant) γH2A(X) that is known to accumulate early-on at sites of DNA damage. In an unperturbed *T. brucei* population, typically ∼10% of cells contained a single focus per nucleus, while the majority of cells had no detectable nuclear γH2A signal (Figures 4A–4C), as expected (Glover and Horn, 2012). Twenty-four hours after PPL2 knockdown, almost all cells contained multiple subnuclear γH2A foci (Figure 4A), and similar results were obtained following PriL knockdown (Figure 4B). This increase in γH2A foci following either PPL2 or PriL knockdown strongly suggests the accumulation of nuclear DNA damage in these cells. In contrast, no substantial increase in the proportion of cells with γH2A foci was observed in PPL1 knockdown cells (Figure 4C). We also saw no increase in sensitivity to methanesulfonate (MMS) following PPL1 knockdown (data not shown).

A DNA repair enzyme known to assemble into foci following DNA damage is the recombinase Rad51. Rad51 plays a central role in homologous recombination, which repairs DNA double-strand breaks in addition to supporting genome replication. In unperturbed *T. brucei* cells, a single Rad51 focus was typically detected in ∼1% of nuclei (Figures 4D and 4E), as expected (Proudfoot and McCulloch, 2005; Glover et al., 2008). Following 24 hr of PPL2 knockdown, almost 90% of cells displayed multiple subnuclear Rad51 foci (Figure 4D). Indeed, almost 15% of cells scored positive for Rad51 foci after only 8 hr of PPL2 knockdown (Figure 4D). PriL knockdown also resulted in the assembly of Rad51 foci, but to a lesser extent. In this case, ∼30% of cells contained multiple subnuclear foci after 24 hr of knockdown (Figure 4E). The accumulation of γH2A and Rad51 foci following PPL2 knockdown indicates the accumulation of DNA damage in the vast majority of these cells. A terminal deoxynucleotidyl transferase dUTP nick end-labeling (TUNEL) assay also revealed increased nuclear DNA damage following PPL2 knockdown (Figure S3). We conclude that accumulation of irreparable DNA damage is the likely cause of cell-cycle arrest and cell death following PPL2 knockdown.

### TbPPL1 Is a DNA-Directed Primase-Polymerase; TbPPL2 Is a DNA Polymerases

AEPs have been shown to be versatile nucleotidyl transferases in vitro (Lao-Sirieix et al., 2005; Pitcher et al., 2007). Since *T. brucei* PPL1 and PPL2 contain the conserved motifs and residues of the AEP superfamily (Figure 1A), we wanted to determine their enzymatic activity. The coding sequences of PPL1 and PPL2 were cloned into an *E. coli* expression vector and the recombinant proteins subsequently purified from *E. coli* containing the expression construct (Figure S4). To be certain that the observed enzymatic activities were intrinsic properties of either PPL1 or PPL2, catalytic mutants were generated in motif I of the AEP domain, which is predicted to be required for binding of divalent metal ions that are essential for AEP activity (D165A, E167A in PPL1 [PPL1 AxA] and D193A D195A in PPL2 [PPL2 AxA]). These PPL1 and PPL2 catalytic mutants were expressed and purified in a similar manner to their wild-type counterparts.

**Figure 5:**
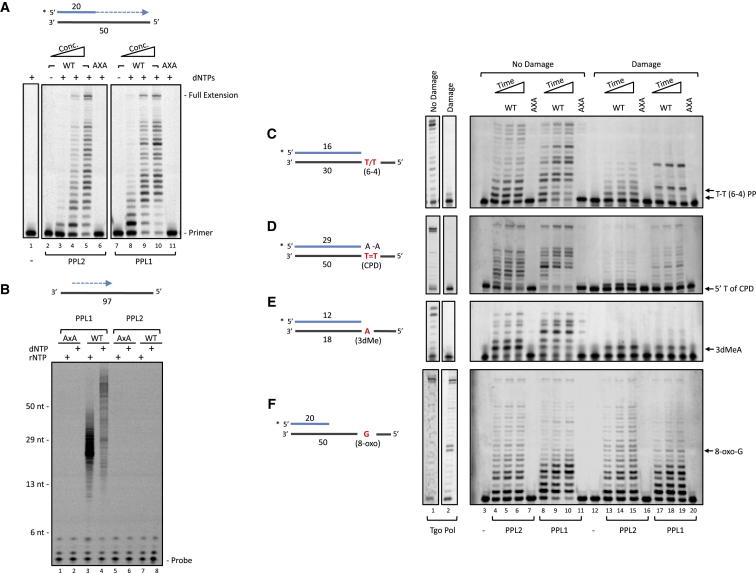
TbPPL1 and TbPPL2 Are DNA-Dependent Translesion DNA Polymerases (A) DNA synthesis by His-tagged TbPPL1 and TbPPL2. Primer template substrate (20 nM) containing a 5′ fluorescent label on the primer strand and dNTPs (200 μM) were incubated without (−) or with wild-type (WT) TbPPL1 or TbPPL2 (50, 125, 250 nM) or catalytic (AxA) mutants (250 nM) for 30 min at 37°C. (B) Primer synthesis by TbPPL1 and TbPPL2. A single-stranded DNA template (500 nM) of variable sequence was incubated with either dNTPs or rNTPs (500 μM) and TbPPL1 or TbPPL2 (1 μM) for 2 hr at 37°C, and products were detected as described in Experimental Procedures. (C–F) DNA synthesis by TbPPL1 and TbPPL2 on templates containing a T-T pyrimidine (6-4) pyrimidone photoproduct (6-4 PP) (C), a T-T *cis-syn* cyclobutane pyrimidine dimer (CPD) (D), a 3-deaza 3-methyladenine (3dMeA) (E), and an 8-oxo-2′-deoxyguanosine (8-oxo-G) (F). (C), (E), and (F) contain substrates with primer termini 3′ of the templated lesion, thereby testing read-through of the lesion, while the primer in (D) contains two 3′ terminal dA residues annealed opposite the CPD, thereby testing extension from the lesion. Primer extensions performed as in (A) except with 125 nM TbPPL1 or TbPPL2 for 10, 20, and 30 min or just a single 30 min time point. As a control the archaeal family-B replicase Tgo-Pol exo^−^ (100 nM) was used.

We began by determining if PPL1 and PPL2 were capable of DNA-dependent DNA polymerase activity using primer extension assays. Primer extensions employ an oligonucleotide template that is annealed to a shorter labeled DNA primer, yielding a double-stranded DNA with a 5′ overhang. Addition of individual bases to the labeled primer is visible as a ladder of products with decreased electrophoretic mobility. Extension of the labeled primer was dependent on both the presence of either PPL1 or PPL2 and deoxynucleotides (dNTPs) (Figure 5A). No extension was observed with the catalytic mutants, confirming the activity was intrinsic to PPL1 and PPL2 (Figure 5A, lanes 6 and 11).

We also tested whether PPL1 and PPL2 were capable of de novo RNA/DNA synthesis on a range of single-stranded DNA templates using a fluorescent primase assay. Primases require an initiation site for dinucleotide formation, and the minimum required by eukaryotic replicative primases is a templated pyrimidine (Frick and Richardson, 2001), and therefore we initially assayed for priming on a d(TCC) repeating template. While no primer synthesis was observed from PPL2, PPL1 was capable of synthesizing RNA primers up to 50 nucleotides in length, and this activity was abolished in the catalytic mutant (Figure S5A). Notably, in further experiments using homopolymer templates, PPL1 was capable of synthesizing DNA primers on a poly(dT) template, but not any other homopolymer, demonstrating a specific requirement for templated dT residues for dideoxynucleotide formation (Figure S5B). Finally, we tested primase activity on a 97-mer of variable DNA sequence and confirmed that while PPL1 was capable of synthesizing both RNA and DNA primers, no activity was observed from PPL2 (Figure 5B).

### TbPPL1 and TbPPL2 Are Translesion Synthesis DNA Polymerases

We have shown that PPL2 plays an essential role in *T. brucei* that is required for cell-cycle progression from late S phase into mitosis. A possible function of PPL2 could be postreplication repair; a DNA damage tolerance process that occurs after replication fork progression and requires specialized DNA polymerases (Ulrich, 2011). In line with a role in DNA damage tolerance would be PPL2’s ability to catalyze TLS. To test this hypothesis in vitro, we employed primer extension assays using oligonucleotide templates containing site-specific DNA lesions.

Two well-characterized replicative polymerase-blocking lesions are produced when DNA is exposed to ultraviolet (UV) light: cyclo-butane pyrimidine dimers (CPDs) and pyrimidine (6-4) pyrimidone photoproducts (Rastogi et al., 2010). We began by testing the ability of PPL1 and PPL2 to replicate DNA containing thymine-thymine (T-T) UV photoproducts. As a control we used an archaeal replicative (family-B) polymerase Tgo Pol (exo^−^) (Hopfner et al., 1999). Tgo Pol could fully extend the primer annealed to the undamaged templates, but was completely incapable of doing so in the presence of either a templated CPD or 6-4 photoproduct, stalling opposite the 3′ T of the lesion (Figures 5C, lane 2 and S6A, lane 7). This confirms these lesions block replicative DNA polymerases. Although PPL1 and PPL2 could not fully replicate a T-T CPD-containing template, stalling prior to the lesion (Figure S6A), they could incorporate bases opposite a T-T 6-4 photoproduct, and further, could extend from these termini by a total of six nucleotides (Figure 5C). The inability of the catalytic mutants to extend the primer (Figure 5C, lanes 16 and 20) confirmed this TLS activity was intrinsic to PPL1 and PPL2. We next asked whether PPL1 or PPL2-dependent bypass of a 6-4 photoproduct was error free or mutagenic using primer extension assays with single dNTPs. In the absence of a templated T-T 6-4 photoproduct, both PPL1 and PPL2 correctly incorporated two As opposite the undamaged TT; however, in the presence of the lesion, a T was incorporated opposite the 3′ T of the lesion, and G or C incorporated opposite the 5′ T of the lesion (Figure S6B). Thus, PPL1 and PPL2 catalyze error-prone bypass of a 6-4 photoproduct.

As TLS can be a cooperative process between two DNA polymerases, with one polymerase first incorporating nucleotides opposite the lesion and a second polymerase extending from this mismatched terminus (Sale et al., 2012), we next tested whether PPL1 and PPL2 could function as an extender in CPD bypass using a substrate containing two A residues at the 3′ terminus annealed opposite the templated T-T CPD. Tgo Pol was completely incapable of extending the primer annealed to the CPD template (Figure 5D, lane 2). In contrast, PPL1 and PPL2 were able to fully extend this primer, and although this was less efficient than with an undamaged template, a substantial amount of extended product was observed (Figure 5D), and this bypass activity was abolished in the catalytic mutants (Figure 5D, lanes 16 and 20).

We also investigated whether PPL1 and PPL2 could bypass other DNA lesions. Among those tested were the cytotoxic 3-methyl adenine (3MeA) lesion that is produced in DNA by the action of S_N_2 methylating agents, the major oxidative lesion 8-oxoguanine (8-oxoG), and an abasic site, which can occur spontaneously or as an intermediate of the base excision repair pathway. Tgo-Pol was unable to bypass a stable 3-deaza analog of 3MeA (3dMeA; Plosky et al., 2008) or an abasic site (Figure 5E, lane 2, and data not shown), consistent with these lesions blocking cellular DNA replicases, but could bypass an 8-oxoG, although stalling was visible at the lesion (Figure 5F, lane 2). PPL1 and PPL2 could incorporate a single nucleotide opposite a templated 3dMeA but could not extend further from this terminus (Figure 5E), and both enzymes correctly inserted T opposite the lesion (Figure S6C). In contrast, PPL1 and PPL2 were incapable of bypassing an abasic site, stalling prior to the lesion (Figure S6D), and could not extend from an A opposite the lesion (Figure S6E). Notably, PPL1 and PPL2 could bypass a templated 8-oxoG (Figure 5F), and both enzymes equally misincorporated A or correctly incorporated C opposite the lesion (Figure S6F). In conclusion, we have demonstrated that *T. brucei* PPL1 and PPL2 are capable of TLS of replication-blocking DNA lesions, including the highly distorting 6-4 photoproduct. Taken together with the cellular data, our results indicate that PPL2 plays an essential role in postreplication DNA damage tolerance in *T. brucei*.

### TbPPL2 Relocalizes to Repair Foci Following DNA Damage

**Figure 6:**
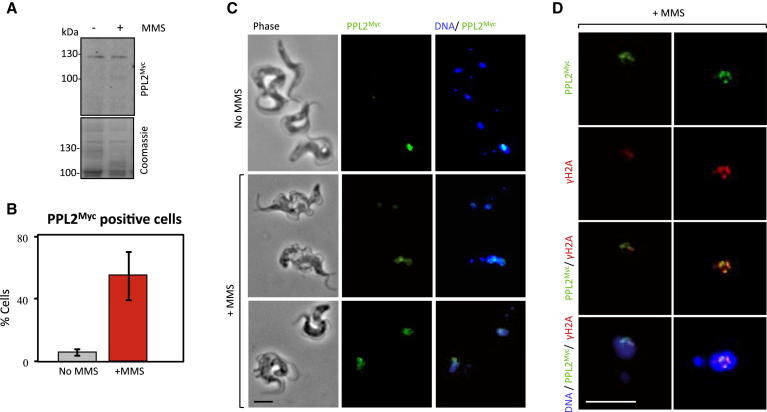
TbPPL2^Myc^ Accumulates into DNA Repair Foci TbPPL2^Myc^ cells were grown in the presence or absence of 0.0003% MMS for 24 hr. (A) Western blot analysis with anti-cMyc and cell lysates prepared from MMS-treated cells. The Coomassie-stained gel serves as a loading control. (B) Immunofluorescence analysis of MMS-treated cells with anti-cMyc; the proportion of cells with TbPPL2^Myc^ staining was determined. n = 200; error bars, standard deviation. (C) Representative images of cells scored in (B). Scale bar is 5 μm. (D) Immunofluorescence analysis of MMS-treated cells with anti-cMyc (for PPL2) and anti-γH2A. Scale bar is 5 μm.

We next looked for further evidence of a role for PPL2 in DNA damage tolerance in vivo. Strains expressing PPL2^Myc^ were treated with the alkylating agent MMS for 24 hr and analyzed by immunofluorescence microscopy. MMS is capable of producing a number of DNA lesions, one of which is the replication-blocking 3MeA, the stable analog of which was bypassed by PPL2 in vitro (Figure 5D). Treatment with MMS did not appear to elicit a major increase in the expression of PPL2^Myc^ (Figure 6A). As described above, PPL2^Myc^ is detected in approximately 6% of unperturbed nuclei (Figure 6B); however, following MMS treatment, a >9-fold increase was observed, with PPL2^Myc^ detected in 55% of nuclei (Figures 6B and 6C). We suspected that the focal accumulation of PPL2^Myc^ represented DNA damage foci, and to test this idea, we examined PPL2^Myc^ and γH2A localization in parallel. This analysis revealed a striking colocalization of PPL2^Myc^ with DNA repair foci (Figure 6D). This redistribution of the PPL2^Myc^ cellular pool, in response to MMS exposure, and to DNA damage foci, clearly establishes a role for PPL2 in the DNA damage response. Taken together, our results indicate that PPL2 is essential for postreplication DNA lesion bypass in African trypanosomes.

## Discussion

PrimPol (CCDC111) is a eukaryotic primase-polymerase originally identified as a member of the AEP superfamily (Iyer et al., 2005). Here, we have identified and characterized two PPLs in one of the earliest diverging eukaryotic organisms that is amenable to genetic manipulation, the pathogen *T. brucei*. While one of the PPL proteins was dispensable for cell proliferation, the second, PPL2, was essential for cell survival.

Several classes of data suggest that PPL2’s essential role occurs during or immediately following DNA replication. PPL2 was detected primarily in the nuclei of G_2_ cells. Depletion of PPL2 results in cells arresting with a ploidy of 4n with an accumulation of DNA damage, as indicated by the assembly of DNA damage signaling (γH2A) and repair (Rad51) foci in most cells. Comparison of this phenotype with cells depleted of PriL, a component of the replicative DNA primase, further supports a distinct role for PPL2 in DNA replication. Depletion of PriL arrests cells in S phase with accumulation of γH2A but not Rad51 foci in most cells. Given that PriL is a component of the canonical DNA primase complex involved in S phase initiation, we expect defects to arise at the replication fork following PriL depletion. It has been reported in *T. brucei* that problems arising during replication result in the assembly of γH2A foci (Glover and Horn, 2012) but not Rad51 foci (Glover et al., 2008), which is consistent with our observations following PriL knockdown. In contrast, PPL2 knockdown results in the vast majority of cells presenting both γH2A and Rad51 foci, consistent with a DNA repair defect that occurs downstream of PriL function. Together, our data are in line with a role for PPL2 in postreplication damage tolerance, also called postreplication repair. Consistent with this, PPL2 was capable of TLS of replication blocking lesions in vitro and, in agreement with a damage response role, following MMS treatment PPL2 relocalized to sites of DNA damage in vivo.

**Figure 7:**
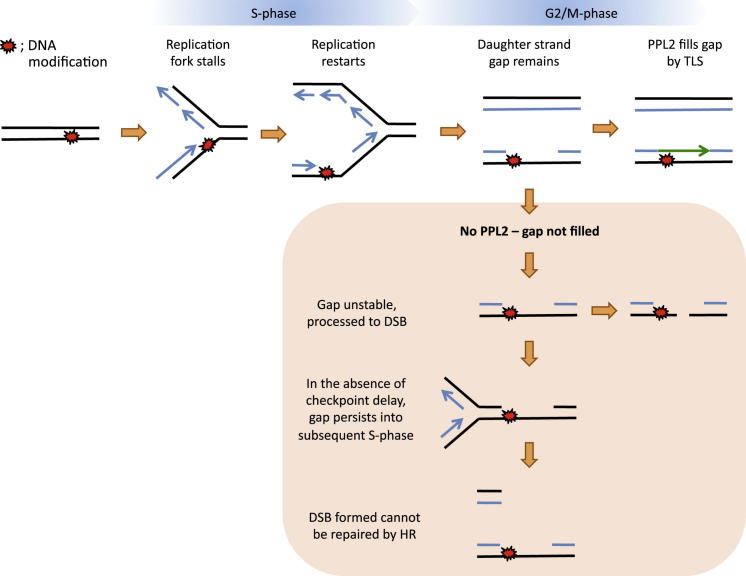
Proposed Model for Role of TbPPL2 in Cell Proliferation In all cells during every cell cycle, the DNA replication machinery will inevitably encounter abnormal or damaged DNA templates that result in the replication fork stalling. Replication can proceed despite these damaged templates by replication restart (shown on leading strand) or convergence of adjacent replicons. This will create a single-stranded gap opposite the damaged template. The proposed role of *T. brucei* PPL2 is to fill in these gaps using its TLS activity (green line), thus restoring the abnormal template in double-stranded DNA and facilitating its later repair. If PPL2-dependent damage tolerance does not operate, the postreplication gaps will persist and eventually form cytotoxic double-strand breaks (DSBs) in the DNA.

Thus, we propose the following model for the function of PPL2 (Figure 7). In every cell during each cell cycle, despite proficient DNA repair mechanisms, the replication machinery will inevitably encounter abnormal or damaged DNA templates, which can arise from environmental insults or endogenous processes. This will result in stalling of the replication fork, and so several mechanisms exist to overcome this problem. The DNA lesion can either be bypassed directly at the fork by TLS or template switching, or it can be bypassed in a postreplicative manner. Replication initiated from adjacent origins can converge on the stalled fork allowing completion of the bulk of DNA synthesis, and replication can reinitiate downstream of the lesion by Okazaki fragment synthesis on the lagging strand and by repriming on the leading strand (Heller and Marians, 2006; Lopes et al., 2006). This will result in a daughter strand with a single-stranded gap opposite the DNA lesion (postreplication gap). We propose that *T. brucei* PPL2 is responsible for filling these gaps, possibly using its TLS activity (Figure 7), thereby restoring the DNA double helix prior to cell division. The phenomenon of postreplication repair has long been documented in bacteria, yeast, and humans (Rupp and Howard-Flanders, 1968; Lehmann, 1972; di Caprio and Cox, 1981). More recently, it has been elegantly demonstrated in budding yeast that DNA damage tolerance can be completely separate from DNA replication and still be fully operational (Karras and Jentsch, 2010; Daigaku et al., 2010). In fact, in both budding yeast and cultured human cells, there is a preference for bypassing some DNA damage following the bulk of DNA synthesis rather than during DNA replication (Callegari and Kelly, 2006; Diamant et al., 2012). In support of this, the Y-family polymerase REV1 in budding yeast is mainly expressed in G_2_ cells, which is when it is suggested to perform its critical DNA damage bypass function (Waters and Walker, 2006). In some instances, damage bypass in G_2_ has been shown to be more mutagenic than bypass during S phase (Diamant et al., 2012). Indeed, PPL2 could contribute to the genetic diversity of *T. brucei* through mutagenic bypass of DNA damage.

According to our model, in cells depleted of PPL2, the postreplication gaps would remain unfilled (Figure 7). Postreplication gaps have been shown to contribute to checkpoint activation, leading to a G_2_ arrest (Karras and Jentsch, 2010; Daigaku et al., 2010; Callegari et al., 2010), which could be consistent with the 4n arrest observed following PPL2 knockdown. Also, postreplication gaps would be subject to further DNA metabolic processes and lead to double-strand breaks (Elvers et al., 2011), in line with γH2A and Rad51 foci observed in the absence of PPL2. The phenotype observed following PPL2 knockdown has striking similarity to UV-exposed *REV1*^*−/−*^ or *REV3L*^*−/−*^ mouse embryonic fibroblasts, which are capable of almost completely replicating their DNA, but single-stranded gaps remain, encompassing the UV photoproduct, which causes these cells to irreversibly arrest in G_2_ (Jansen et al., 2009a, 2009b). One major difference between the studies referenced thus far and the observations reported here is that the phenotype resulting from PPL2 knockdown occurs in the absence of external challenges to DNA. This is intriguing, as only one other TLS polymerase has been reported to be essential for normal cell proliferation, that being mammalian Pol ζ (Lange et al., 2012). In the absence of Pol ζ, primary mouse fibroblasts accumulate strand breaks and chromosome aberrations during a single cell cycle and, following further cell divisions, ultimately proceed through apoptosis (Lange et al., 2012).

The naturally occurring DNA damage responsible for the severe phenotype observed in PPL2-depleted cells is unknown. PPL2 can bypass replication-blocking lesions, such as UV photoproducts, but it is very unlikely that UV photoproducts occur naturally in these blood-borne parasites. However, what we have demonstrated is the inherent adaptability of the PPL catalytic center to accept helix-distorting lesions, and so it is predictable that PPL proteins could bypass other DNA lesions. Common sources of endogenous DNA damage include reactive oxygen species, which was shown to be a contributing factor to Pol ζ’s essential role in mammalian cells (Lange et al., 2012). Also, misincorporation of ribonucleotides into genomic DNA by the replicative polymerases has been shown to pose a significant problem to replication, and it has been demonstrated in budding yeast that a Pol ζ-dependent postreplication repair mechanism is responsible for correcting these errors (Lazzaro et al., 2012). It is also worth considering that *T. brucei* may be more susceptible to DNA damage during DNA replication than other eukaryotes, given the relative scarcity of active replication origins in their megabase chromosomes (Tiengwe et al., 2012b), which may put increased importance on DNA damage repair and tolerance mechanisms in order to ensure genome duplication. Additionally, in trypanosomatid nuclear genomes, about 1% of thymines are modified to yield DNA Base J, which was recently demonstrated to block transcription in the trypanosomatid *Leishmania* (van Luenen et al., 2012). It is conceivable that Base J may block replicative DNA polymerases, and therefore specific flexible DNA polymerases may be employed to replicate Base J-containing DNA. TLS polymerases are also important for replicating structured DNA and difficult-to-replicate regions such as chromosomal fragile sites (Bétous et al., 2009; Rey et al., 2009). Thus, PPL2 could be required for correcting errors that accumulate during DNA replication and/or for replicating particular regions containing structured DNA. The finding that PPL2 is essential in *T. brucei* suggests a lack of redundancy in terms of the role(s) fulfilled by this DNA replication activity. Indeed, PPL1 clearly fails to complement for the PPL2 defect.

DNA replication enzymes are potential targets for antitrypanosomal drugs. The phenotype observed following PriL depletion was both rapid and lethal, as was also the case for PPL2. While other eukaryotes appear to contain only one essential AEP (DNA Primase), trypanosomes have evolved a DNA replication mechanism that requires two essential AEPs: the replicative DNA primase to initiate DNA replication, and PPL2, a flexible DNA polymerase, which is essential to complete DNA replication. Taken together, our findings demonstrate that *T. brucei* is an excellent model system in which to investigate DNA replication by primases and polymerases. Specifically, we have employed this system to identify an essential TLS-bypass activity that acts downstream of the canonical replicative primase. Our results emphasize the importance of DNA damage tolerance during DNA replication and have major implications for related processes and for the role of related enzymes in other eukaryotes.

## Experimental Procedures

### *T. brucei* Strains

Bloodstream form *T. brucei*, Lister 427, MiTat 1.2, clone 221a, and derivatives including the 2T1 strain were maintained as previously described (Alsford and Horn, 2008). For growth analysis, *T. brucei* were seeded at a density of 1 × 10^5^/ml and split back to 1 × 10^5^/ml every 24 hr where necessary. Counts were carried out using a hemocytometer. For RNAi induction, tetracycline (Sigma) was applied at 1 μg/ml. MMS (Sigma) was applied to *T. brucei* cultures at 0.0003% for 24 hr.

### *T. brucei* Constructs

The *PPL1*, *PPL2*, and *PriL* genes used in this study were derived from Lister 427 *T. brucei* strain. Gene fragments were amplified by PCR from genomic DNA using Phusion high-fidelity DNA polymerase (Finnzymes) with specific primer pairs and inserted into the relevant vector (see Table S1): pRPa^iSL^ for Tet-inducible RNAi and pNAT^x12M^ for native-allele tagging. Plasmid linearization, stable transformation, and clone selection were carried out as previously described (Alsford and Horn, 2008).

### Protein Analysis

Cells were lysed in SDS loading buffer and were separated on SDS polyacrylamide gels and stained with Coomassie blue or subjected to western blot analysis. We used mouse anti-Myc at 1:4,000 and goat anti-mouse IgG HRP (Bio-Rad) was used at a 1:2,000 dilution with a chemiluminescent kit (GE Healthcare), according to the manufacturer’s instructions.

### Cell-Cycle Analysis and Immunofluorescence Microscopy

For flow cytometry, 1 × 10^6^ cells were fixed in 70% methanol, 30% PBS and incubated at 4°C overnight. Cells were washed in PBS, then resuspended in PBS containing 10 μg/ml propidium iodide and 10 μg/ml RNase A and incubated at 37°C for 45 min. A Becton Dickinson FACScalibur was used to analyze samples with CellQuest software and detector FL2-A with an Amp gain value of 1.75. Data analysis was carried out using FlowJo (Treestar). For immunofluorescence microscopy, cells were labeled using a standard protocol with rabbit anti-γH2A primary antisera (Glover and Horn, 2012), mouse anti-Myc primary antisera (1:100 dilution; Source BioScience) or rabbit anti-RAD51 primary antisera (1:250 dilution; Glover et al., 2008) and fluorescein or rhodamine-conjugated goat anti-rabbit or anti-mouse secondary antibodies (Pierce). TUNEL was performed using the Fluorescein In Situ Cell Death Detection Kit (Roche) according to the manufacturer’s instructions. Cells were mounted in VectaShield (Vector Laboratories) containing DAPI. All counts for the quantitative analysis of cell-cycle phases or proportions of cells with Myc, RAD51, and/or γH2A foci were carried out by two of us. Images were captured using a Nikon Eclipse E600 epifluorescence microscope in conjunction with a Coolsnap FX (Photometrics) charge-coupled device (CCD) camera and processed in Metamorph 5.0 (Photometrics).

### Cloning and Protein Purification

The *PPL1* and *PPL2* genes were amplified by PCR from Lister 427 *T. brucei* strain genomic DNA using Phusion high-fidelity DNA polymerase with specific primer pairs (Table S1) and inserted into pET28a (Novagen). Site-directed mutagenesis to create catalytic dead PPL1 and PPL2 was performed also using Phusion high-fidelity DNA polymerase and complementary overlapping primers containing the desired mutation (Table S1). For purification of PPL1 and PPL2, constructs encoding the 6-Histidine-tagged WT or AxA PPL1 or PPL2 were first transformed into *E. coli* B843 DE3 pLysS (B834s), and the recombinant proteins were subsequently purified by nickel (Ni^2+^)-NTA (QIAGEN) and Heparin (GE Healthcare) chromatography columns (see Supplemental Experimental Procedures). Archaeal family-B DNA polymerase from *T. gorgonarius* (Tgo PolB exo^−^) was prepared as described by Jozwiakowski and Connolly (2011).

### Primer Extension Assays

DNA primers containing a 5′ hexachlorofluorescein (Hex) label and nondamaged and damaged DNA templates are detailed in Table S2. All were purchased from ATDBio except the 6-4 PP and 3dMeA templates, which were gifts from Alan Lehmann (GDSC, Brighton) and Roger Woodgate (NIH, Maryland), respectively. Primer extension assays were performed as described previously (Jozwiakowski and Connolly, 2011). A typical reaction contained 10 mM Bis-Tris-Propane-HCl (pH 7), 10 mM MgCl_2_, 50 mM NaCl, 20 nM primer-template substrate, 200 μM dNTPs (Roche), and 100 nM recombinant PPL1 or PPL2. Following a 10–30 min incubation at 37°C, the reaction was quenched in 20 μl 2× stop buffer (95% formamide, 40 mM EDTA) and incubated at 90°C for 2 min. The products were resolved on 15% polyacrylamide/7M urea gel by electrophoresis and scanned to detect the fluorescent label on a FUJI FLA-1500 scanner.

### Primase Assay

The fluorescent primase assay was performed in three steps and is described in detail in the Supplemental Experimental Procedures. Briefly, a typical priming reaction contains 10 mM Bis-Tris-Propane-HCl (pH 7), 10 mM MgCl_2_, 50 mM NaCl, 500 nM DNA template (with a 5′ biotin moiety; see Table S3), 500 μM dNTPs (Roche) or rNTPs (Invitrogen), and 1 μM recombinant TbPPL1 or TbPPL2. The reaction products are labeled using 15 μM FAM-6-dATP (Jena-Biosciences) and 0.2 U of klenow-Taq. The labeled products are purified using the 5′ biotin moiety of the template, boiled to liberate the FAM-labeled primers, and then resolved on a 15% polyacrylamide/7M urea gel before fluorescent detection.

## Supplemental Information

Document S1. Figures S1–S6, Tables S1–S3, and Supplemental Experimental Procedures
