# Supplementary material for: PPL2 Translesion Polymerase Is Essential for the Completion of Chromosomal DNA Replication in the African Trypanosome
Source: Mol Cell. 2013 Nov 21;52(4):554–65. doi: 10.1016/j.molcel.2013.10.034 (PMC3898837; doi:10.1016/j.molcel.2013.10.034)
Supplement: Document S1. Figures S1–S6, Tables S1–S3, and Supplemental Experimental Procedures [file mmc1.pdf]

Molecular Cell, Volume *52*

Supplemental Information

PPL2 Translesion Polymerase Is Essential for the Completion of Chromosomal DNA Replication in the African Trypanosome

Sean G. Rudd, Lucy Glover, Stanislaw K. Jozwiakowski, David Horn, and Aidan J. Doherty

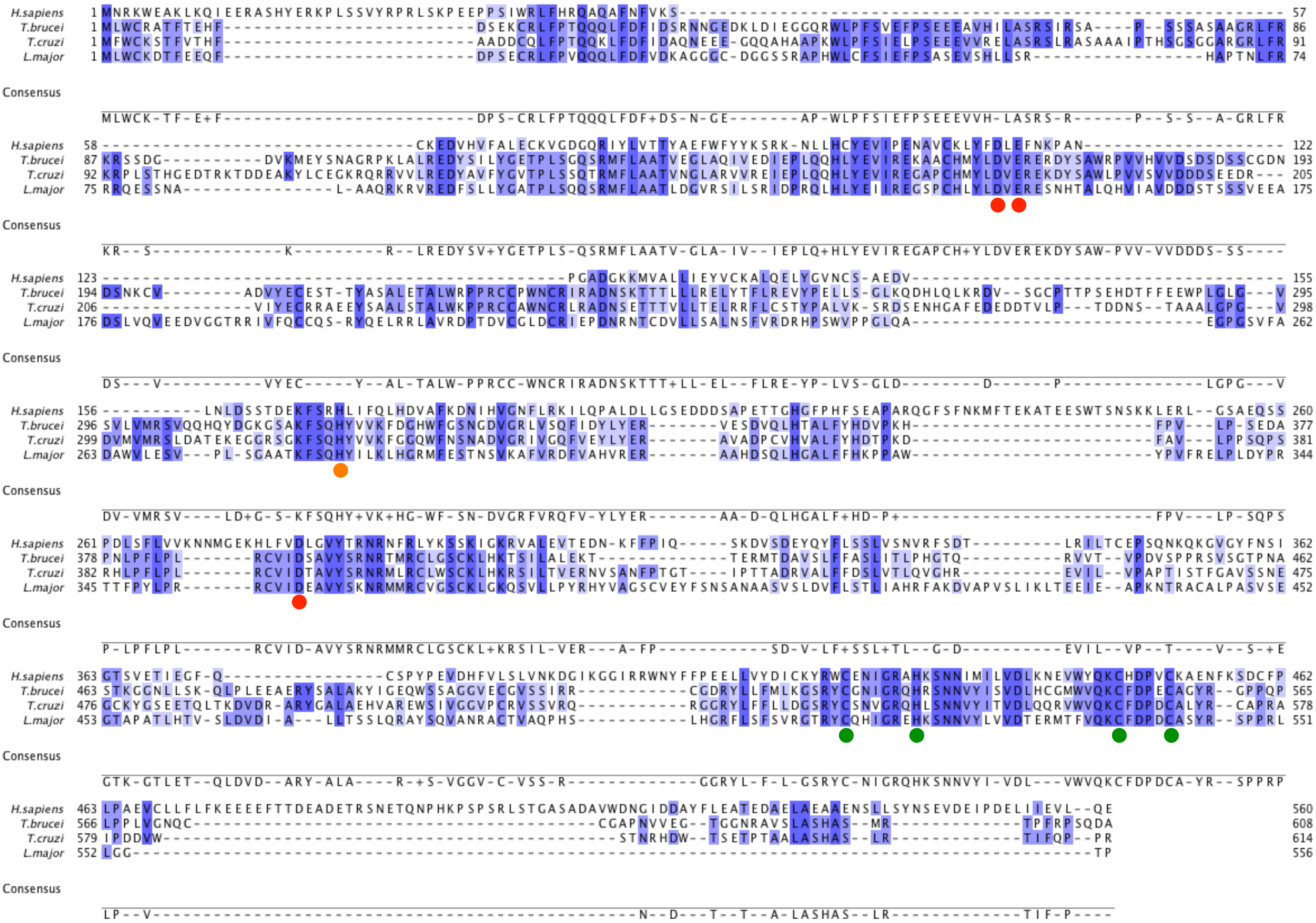

Figure S1. Sequence alignment of trypanosomatid PrimPol-like 1 family (linked with Figure 1)
Multiple sequence alignment of human PrimPol and the *T. brucei*, *T. cruzi*, and *L. major* PPL1 proteins. Predicted essential residues in the AEP domain are indicated, residues in motifs I and III (red circles) required for magnesium ion binding, motif II (orange circle) for nucleotide binding. Residues required for chelation of zinc in the UL52 zinc finger are also indicated (green circles).

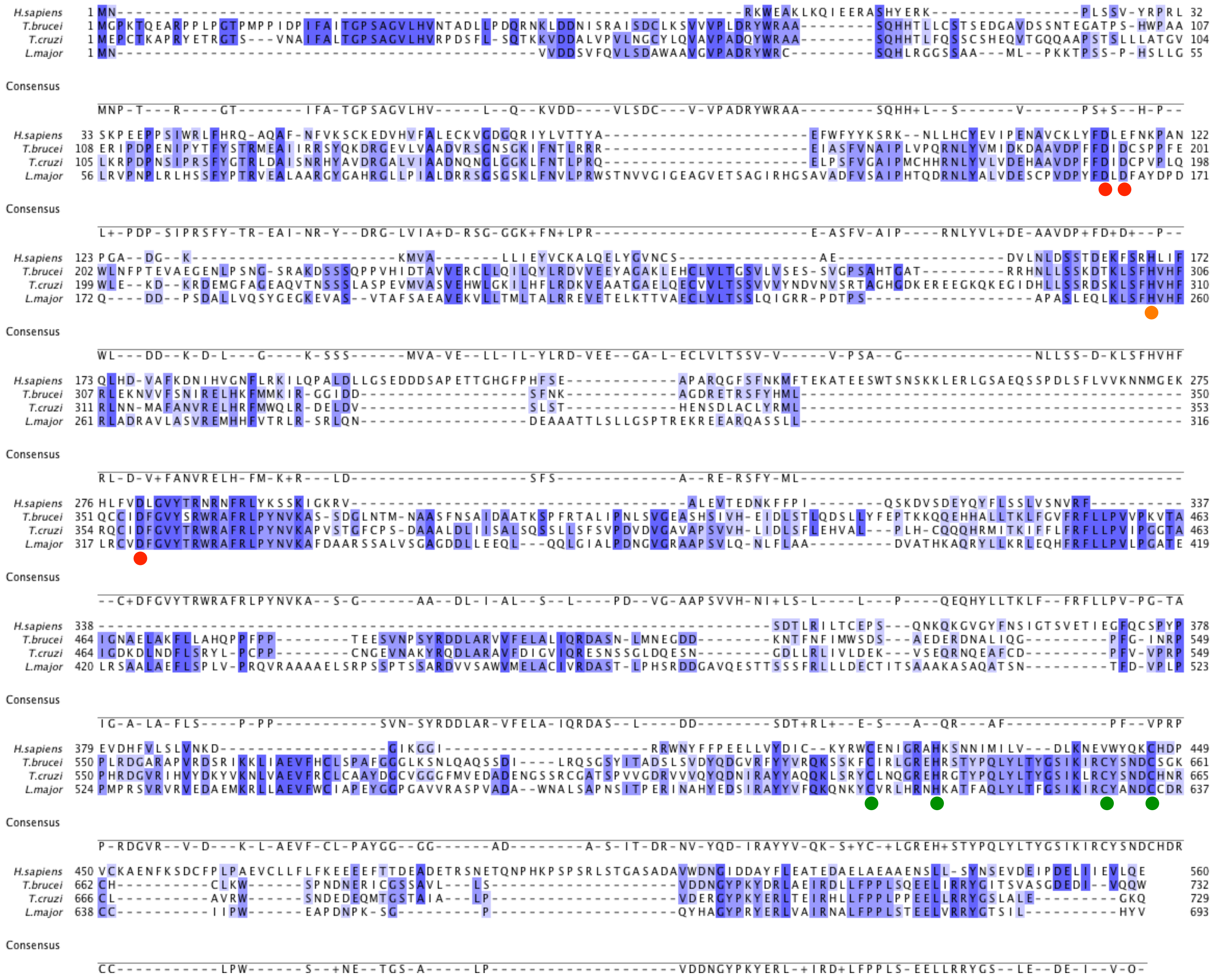

Figure S2. Sequence alignment of trypanosomatid PrimPol-like 2 family (linked with Figure 1) Multiple sequence alignment of human PrimPol and the *T. brucei*, *T. cruzi*, and *L. major* PPL2 proteins. Predicted essential residues in the AEP domain are indicated, residues in motifs I and III (red circles) required for magnesium ion binding, motif II (orange circle) for nucleotide binding. Residues required for chelation of zinc in the UL52 zinc finger are also indicated (green circles).

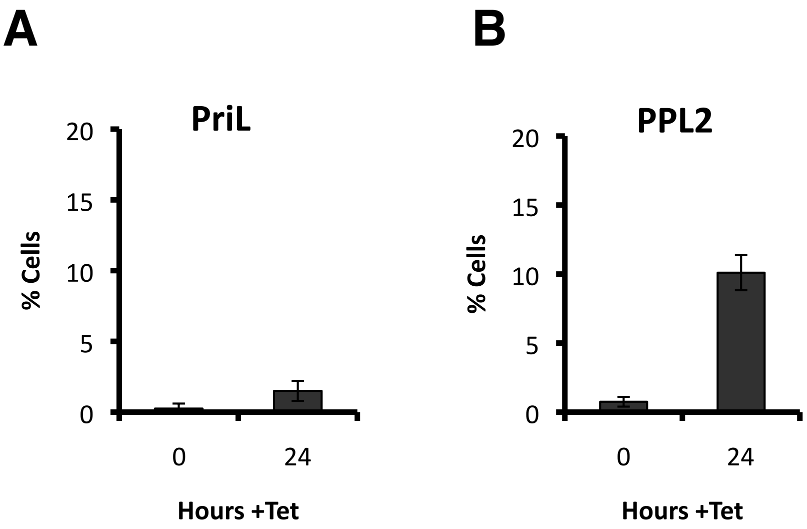

Figure S3. TbPPL2 knockdown increases nuclear TUNEL staining (linked with Figure 4)
Cultures of TbPriL (**A**) and TbPPL2 (**B**) inducible RNAi strains were grown in the presence or absence of 1 μg/ml tetracycline for 24 hours and then fixed. Cells were permeabilised and free 3' OH ends of DNA were labelled with fluorescein-labelled nucleotides by TdT for 1 hour (TUNEL method). Cells were then counterstained with DAPI and analysed by fluorescence microscopy and the proportion of cells with nuclear TUNEL staining calculated. Two experiments were performed for each RNAi strain, counting at least 200 cells each time. Error bars represent standard deviation.

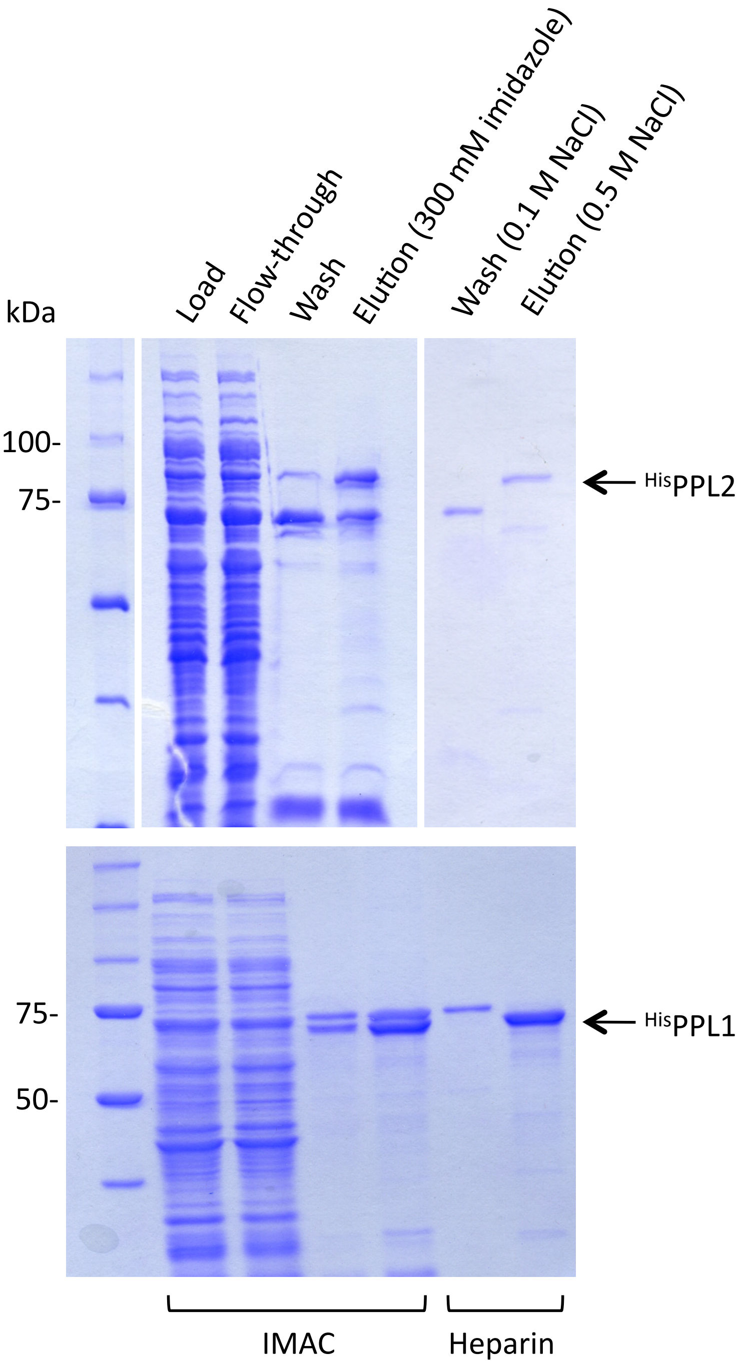

Figure S4. Preparation of recombinant TbPPL1 and 2 (linked with Figure 5)
Recombinant His-TbPPL1 and 2 were purified from over-expressing *E. coli* cell extracts using nickel-chelate and heparin chromatography. Samples from each purification step were analysed by SDS-PAGE and Coomassie blue staining. See Supplementary Experimental Procedures for further detail.

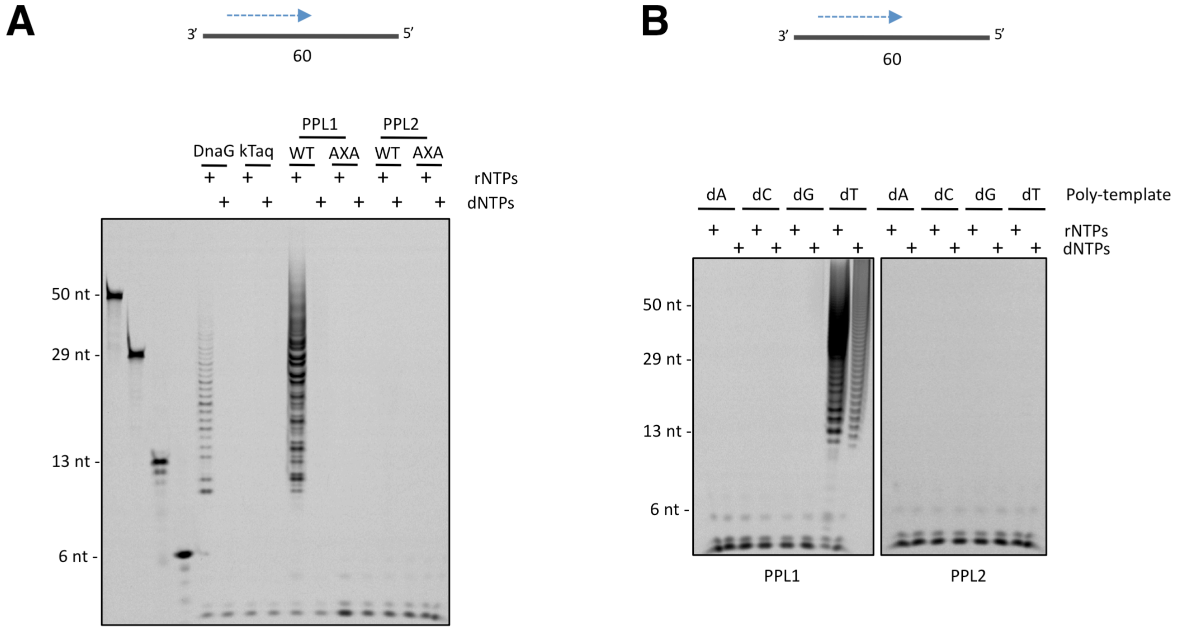

Figure S5. Supplementary primase assays (linked with Figure 5)

Primer synthesis by His-TbPPL1 and 2 on a (**A**) d(TCC)$_{20}$ template or (**B**) dA, dC, dG, dT homopolymer templates as indicated. DNA templates (500 nM) were incubated with either dNTPs or rNTPs (500 µM) and TbPPL1 or 2 (1 µM) for 2 hours at 37°C. Products were detected as described in Experimental Procedures. Recombinant *Bacillus stereothermophilus* DnaG primase (1 µM) was used as a positive control for primer synthesis, and klenow-Taq (kTAQ) DNA polymerase (1 µM), which is used in the primer-labelling step, as a negative control.

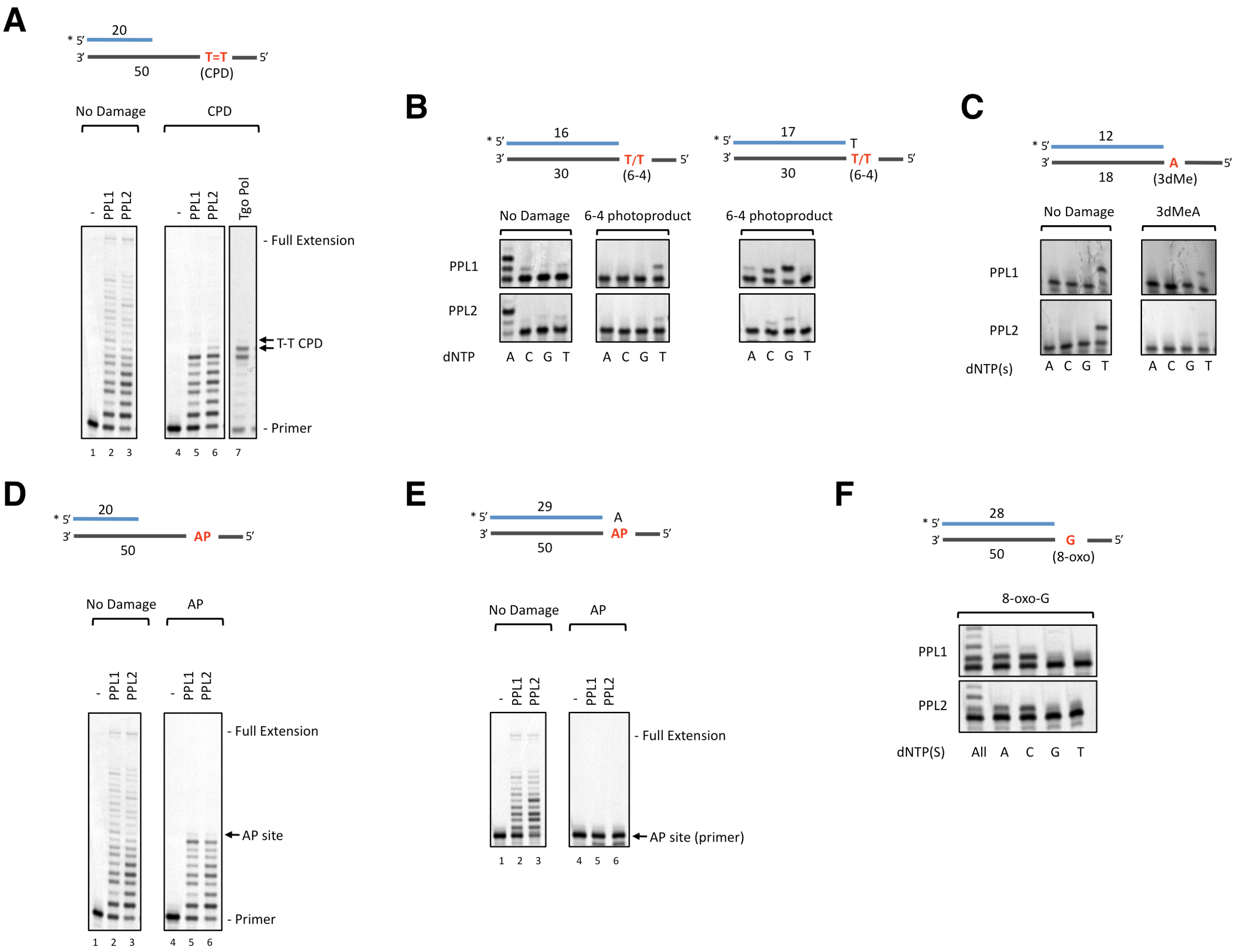

Figure S6. Supplementary primer extension assays with lesion-containing templates (linked with Figure 5)

Primer extensions with substrates containing templated DNA lesions: (**A**) *cis-syn* cyclobutane pyrimidine dimer (CPD), (**B**) pyrimidine (6-4) pyrimidone photoproduct (6-4 PP), (**C**) 3-deaza 3-methyladenine (3dMeA), (**D** and **E**) abasic (AP) site, and (**F**) an 8-oxo-2'-deoxyguanosine (8-oxo-G). Primer template substrates (20 nM) were incubated with dNTP(s) (200 µM) and TbPPL1 or 2 (125 nM) for 30 minutes at 37°C. Each substrate is represented schematically indicating the 5' fluorescent label on the primer strand (asterisk), the location of the templated DNA lesion, and the 3' terminal residues of the primer strand if relevant.

Gene	Purpose	Primer Sequence (5'-3')	Restriction sites	Vector (entry)
PPL1	RNAi	GATC**TCTAGA**GGATCCTGCATCGCTCATTACTTTGC	**XbaI** BamHI	pRPa^{iSL}
		GATC**CTCGAG**GTACCGTTGCGGTGGTCCTCTGTAT	**XhoI** KpnI	
	Tagging	GATCGGCGCGCCGCTTGGTTTGGGCG	AscI	pNAT^{x12M}
		GATCTCTAGAAGCGTCCTGCGACGGAC	XbaI	
	Cloning	GCTCATATGCTCTGGTGTAGGGCAACG	NdeI	pET28a
		CGGATCCCTAAGCGTCCTGCGACG	BamHI	
	AxA mutation	GTATTTGG**cc**GTTG**ct**CGGGAGAGG		
		GTATTTGG**cc**GTTG**ct**CGGGAGAGG		
PPL2	RNAi	GATC**TCTAGA**GGATCCAGACGGCATAATCTGCTCA	**XbaI** BamHI	pRPa^{iSL}
		GATC**CTCGAG**GTACCATGGCTGTTACTTTGGGCAC	**XhoI** KpnI	
	Tagging	GATCAAGCTTCATCATGTGGAGTGATT	HindIII	pNAT^{x12M}
		GATCTCTAGACCACTGTTGGACGATATCT	XbaI	
	Cloning	CGGATCCCTAAGCGTCCTGCGACG	BamHI	pET28a
		CGAATTCTCACCACTGTTGGACGATATC	EcoRI	
	AxA mutation	CCTTTTTTCG**cg**ATAG**c**CTGCAGCCCACCGTTTGAGTGGTTGAATTTTCC		
		GTGGGCTGCAG**g**CTAT**cg**CGAAAAAAGGGTCAACTGCGGCGTCTTTATC		
PriL	RNAi	GATC**TCTAGA**GGATCCTTTTGTTTTCTGCATGAGCG	**XbaI** BamHI	pRPa^{iSL}
		GATC**CTCGAG**GTACCAAGTGTGTCTGCGCAAGATG	**XhoI** KpnI	
	Tagging	GATCAAGCTTCGGCTGGTGCTG	HindIII	pNAT^{x12M}
		GATCTCTAGACAAAGAATCACGCGGACGG	XbaI	

Table S1. Primers used to generate constructs. Restriction sites are underlined or in bold; mutations are in lowercase and in bold.

	Length	Sequence (5'-3')	Modification
**Template**	50	CGCGCAGGGCGCACAACAGCCTTGAAGACCGAACGACCGAACAGACGACA	No damage
	50	CGCGCAGGGCGCACAACAGCCTTGAAGACCGAACGACCGAACAGACGACA	CDP (TT)
	50	CGCGCAGGGCGCACAACAGCCGTGAAGACCGAACGACCGAACAGACGACA	8-oxo-G (G)
	50	CGCGCAGGGCGCACAACAGCC_TGAAGACCGAACGACCGAACAGACGACA	AP site (_)
**Primer** (5' HEX label)	20	TGTCGTCTGTTCGGTCGTTC	
	28	TGTCGTCTGTTCGGTCGTTCGGTCTTCA	
	29	TGTCGTCTGTTCGGTCGTTCGGTCTTCAA	
**Template**	30	CTCGTCAGCATCTTCATCATACAGTCAGTG	No damage
	30	CTCGTCAGCATCTTCATCATACAGTCAGTG	6-4 PP (TT)
**Primer** (5' HEX label)	16	CACTGACTGTATGATG	
	17	CACTGACTGTATGATGT	
**Template**	18	AGACGACGTCCTGTAGCC	No damage
	18	AGACGACGTCCTGTAGCC	3dMeA (A)
**Primer** (5' HEX label)	12	GGCTACAGGACG	

Table S2. Primer and template strands for primer extension assays. DNA lesions in templates are indicated in red text: CPD, cyclobutane pyrimidine dimer; 8-oxo-G, 8-oxo-guanine; AP site, abasic site; 6-4 PP, pyrimidine pyrimidone 6-4 photoproduct; 3dMeA, 3-deaza 3-methyladenine. Primers contain a 5’ hexachlorofluorescein (HEX) label.

	Length	Sequence (5’-3’)
variable	97	ACCGCGAACTTGAATTCTGCCGGGGCTTCTCCCGCCTTTTTTCCCGGCGGCGGGAGAAGTAGATTAACATA TGGCTGAACGACCGAACAGACGACA
d(TCC)	60	TCCTCCTCCTCCTCCTCCTCCTCCTCCTCCTCCTCCTCCTCCTCCTCCTCCTCCTCCTCC
d(A)	60	AAAAAAAAAAAAAAAAAAAAAAAAAAAAAAAAAAAAAAAAAAAAAAAAAAAAAAAAAAAA
d(C)	60	CCCCCCCCCCCCCCCCCCCCCCCCCCCCCCCCCCCCCCCCCCCCCCCCCCCCCCCCCCCC
d(G)	60	GGGGGGGGGGGGGGGGGGGGGGGGGGGGGGGGGGGGGGGGGGGGGGGGGGGGGGGGGGGG
d(T)	60	TTTTTTTTTTTTTTTTTTTTTTTTTTTTTTTTTTTTTTTTTTTTTTTTTTTTTTTTTTTT

Table S3. Substrates for primase assays.

SUPPLEMENTARY EXPERIMENTAL PROCEDURES

Preparation of recombinant TbPPL1 and 2

For purification of TbPPL1 and 2, constructs encoding the 6-Histidine tagged WT or AxA TbPPL1 or 2 were first transformed into *E. coli* B843 DE3 pLysS (B834s). Typically 3 litre cultures supplemented with relevant antibiotics and 100 µM $ZnSO_4$ were grown at 37°C to exponential phase and induced with 1 mM IPTG for 3 hours at 25°C before harvesting. Cell pellets were lysed in immobilised metal affinity chromatography (IMAC) buffer A (50 mM Tris-HCl pH 7.5, 0.5 M NaCl, 30 mM Imidazole, 10% Glycerol, 17 µg/ml PMSF, 34 µg/ml Benzamidine, 5 mM 2-mercaptoethanol), sonicated and cleared by centrifugation. The soluble fraction was loaded onto an IMAC column charged with nickel (Ni^{2+})-NTA resin (Qiagen), washed with buffer A and then 10% IMAC buffer B (same as buffer A except 300 mM Imidazole). TbPPLs were eluted with 100% buffer B (300 mM imidazole), diluted 10-fold with Heparin buffer A (50 mM Tris-HCl pH7.5, 10% Glycerol, 2 mM DTT), and loaded onto a Heparin HiTrap HP chromatography column (GE Healthcare). Following a 10% Heparin buffer B wash (same as buffer A except 1M NaCl), TbPPLs were eluted in 50% B (0.5 M NaCl), concentrated and snap frozen in aliquots to be stored at -80°C.

Flourescent primase assay

Sequences of oligonucleotides used in Table S3. The non-radioactive primase assay was performed in three steps. Typically 1 µM of the tested enzyme or 2 U of klenow-Taq (negative control for the assay; purified as described in Engelke et al., 1990) in 20 µl reaction volume containing 500 nM ssDNA synthetic template with a biotin modification at the 5' end, 500 µM rNTPs (Invitrogen) or 500 µM dNTPs (Roche), 10 mM Bis-Tris-Propane-HCl pH7, 10 mM $MgCl_2$, 50 mM NaCl, was incubated for 2 hours at 37°C. The reaction was then supplemented with 0.2 U of kTaq and 15 µM FAM-6-dATP (Jena-Biosciences) and incubated at 37°C for 45 minutes to allow fluorescent labelling of *de novo* synthesised primers. The primer synthesis/labelling enzymatic reactions were terminated by adding 450 µl of binding-washing (B-W) buffer (10 mM Tris-HCl 8.0, 500 mM NaCl, 10 mM EDTA). The quenched reactions were added to ~30 µl of streptavidin coated beads (500 µl total volume) and mixed on a spinning wheel for 1 hour at 4°C. After capturing the ssDNA templates, the suspension was spun down briefly to sediment the beads. The supernatant was removed and the beads were washed three times with 1 ml of B-W buffer. The beads were then suspended in 20 µl of 8 M UREA 10 mM EDTA and boiled for 3 minutes to liberate the primers. The 20 µl samples were spun down briefly, loaded on 15% polyacrylamide / 7M urea gel and resolved for 1h 45min at 17 watts. After

electrophoresis, gels were scanned for fluorescent signal and products of reaction/labelling of *de novo* synthesised primers were visualised. Recombinant *Bacillus stereothermophilus* DnaG (Bst-DnaG) was a gift from Kenneth Marians (Memorial Sloan-Kettering Cancer Center, New York).

SUPPLEMENTARY REFERENCES

Engelke, D. R., Krikos, A., Bruck, M. E., and Ginsburg, D. (1990). Purification of *Thermus aquaticus* DNA polymerase expressed in *Escherichia coli*. Anal. Biochem. *191*, 396-400.
